# Reference of Temperature and Time during tempering process for non-stoichiometric FTO films

**DOI:** 10.1038/srep15001

**Published:** 2015-10-14

**Authors:** J. K. Yang, B. Liang, M. J. Zhao, Y. Gao, F. C. Zhang, H. L. Zhao

**Affiliations:** 1 State Key Laboratory of Metastable Materials Science and Technology, College of Materials Science and Engineering, Yanshan University, Qinhuangdao 066004, China

## Abstract

In order to enhance the mechanical strength of Low-E glass, Fluorine-doped tin oxide (FTO) films have to be tempered at high temperatures together with glass substrates. The effects of tempering temperature (600 °C ~ 720 °C) and time (150 s ~ 300 s) on the structural and electrical properties of FTO films were investigated. The results show all the films consist of non-stoichiometric, polycrystalline SnO_2_ without detectable amounts of fluoride. 700 °C and 260 s may be the critical tempering temperature and time, respectively. FTO films tempered at 700 °C for 260 s possesses the resistivity of 7.54 × 10^−4^ Ω•cm, the average transmittance in 400 ~ 800 nm of ~80%, and the calculated emissivity of 0.38. Hall mobility of FTO films tempered in this proper condition is mainly limited by the ionized impurity scattering. The value of [O]/[Sn] at the film surface is much higher than the stoichiometric value of 2.0 of pure crystalline SnO_2_.

Due to their high transmittance in visible region, high reflectivity in the IR-region and excellent semi-conducting characteristics, fluorine-doped tin oxide films (SnO_2_: F, FTO) have attracted considerable attention in the energy-saving field, such as architectural glass, thin-film solar cells^1^,^2^,^3^. With the increasing demands of safety glass in many architectural applications, various secondary processing^4^, such as bending/vacuum forming and, most importantly, thermal toughening, were carried out to enhance the mechanical strength to avoid breaking into large and sharp fragments. Therefore, new processes are needed to fabricate FTO films that can withstand these high temperatures without a decrease of performance^5^. A post-heat treatment of the glass at a high temperature (often up to 700 °C) is common to increase the toughness of the glass: a process that is called “glass tempering” in industry. During the tempering process, temperature and time are two crucial parameters to improve mechanical properties. Unfortunately, little is known about the electrical properties of FTO films at such high temperatures. A.F. Khan^6^ found that FTO films obtained a relatively lowest resistivity after post-heated at 400 °C in the region of 350 ~ 550 °C. Q. Gao *et al.*^7^ reported that low-emission glass maintained good functional properties below 580 °C. Our previous work^8^ found the electrical performance of FTO films deteriorated after being tempered at 700 °C. Heating time should also be controlled strictly to obtain good electrical properties. Therefore, more experimental data for the optimization of the tempering process is needed.

Here, our aim in this work was to investigate the effects of tempering temperature and time on the microstructural and electrical properties of FTO films during the tempering process and optimize the tempering process.

## Experimental procedure

FTO films were prepared by atmospheric pressure chemical vapor deposition (APCVD) on glass coated with a barrier layer of SiO_x_C_y_^9^. Monobutyltin trichloride (C_4_H_9_SnC_13_, MBTC) and trifluoro acetic acid (CF_3_COOH, TFA) were used as precursor and dopant, respectively. MBTC (99% purity) and TFA (99% purity) were gasified in a bubble room at 160 °C and 20 °C, respectively. High purity N_2_ was used as the carrier gas and H_2_O was used as activator. The molar content of fluorine was held constant at about ~5% during the doping process. FTO films were deposited on the substrate at a temperature of 650 °C. The reaction is described by the following equation:


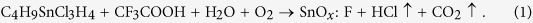


The FTO-coated glass was treated in a tempering furnace at different conditions with a quench pressure of 270 kPa and quench time of 110 s, as listed in Table 1.

The crystal structure were determined using X-ray diffraction (D/MAX-rB) with Cu K_α_ radiation (λ = 0.15406 nm). The optical transmittance spectra were performed in the 190 ~ 900 nm wavelength range using a UV-visible double-beam spectrophotometer (UV 1900). The four-point probe method was employed to measure the electrical properties (Keithley 2400). The electrical resistivity *ρ* can be measured directly and determined as: *ρ* = (*V*·A)/(*l*·*I*), where *V* is the measured potential drop across the sample, *I* is the current through the sample, A is the cross sectional area, and *l* is the separation of the voltage leads. The Hall coefficient at *T* = 300 K is carried out in magnetic fields and electric fields which are perpendicular to each other. The Hall coefficient *R*_H_ can be defined from the relationship: *R*_H_ = (*V*_H_·A)/(*I*·*l*·*B*), where *V*_H_ is the Hall potential, and *B* is the applied magnetic field. The mobility *μ* and carrier concentration *n* can be calculated from Hall coefficient *R*_H_ and resistivity *ρ* as follows: *μ* = |*R*_H_|/*ρ*, *n* = 1/e·|*R*_H_|.

The chemical states of oxygen and tin and the element distribution in films were examined using X-ray photoelectron spectroscopy (XPS, ESCALAB250, Thermo). K_α_ radiation of an Al anode (*hυ* = 1486.6 eV) was used as excitation source. The X-ray gun was operated at 13 kV and 250 W. In order to reduce the effects of Ar^+^ ion etching on the binding energy of electrons, low-energy Ar^+^ ions were used for etching of FTO films. The work pressure in the analysis chamber was 1 × 10^−7^ mbar under irradiation. The C1*s* binding energy of residual carbon present on the film surface, positioned at 285.0 eV, was used to calibrate all the reported binding energy data. XPS data was analyzed using the XPS Peak Fitting Program (version 4.1)^10^. Quantification of the relative concentration of the elements in FTO films was based on the area under the curve of the XPS O1*s* and Sn3*d*_5/2_ peaks and the content was calculated using the atomic sensitivity factor analytical procedure according to the following equation^11^:


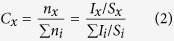


where *C*_*x*_ is the element content, *I*_i_ is the integrated intensity of the element in the XPS spectrum, *S*_i_ is the atomic sensitivity factor, *S*_Sn_ = 4.095, *S*_O_ = 0.711 and *S*_F_ = 1.0.

## Results and Discussions

**Figure 1:**
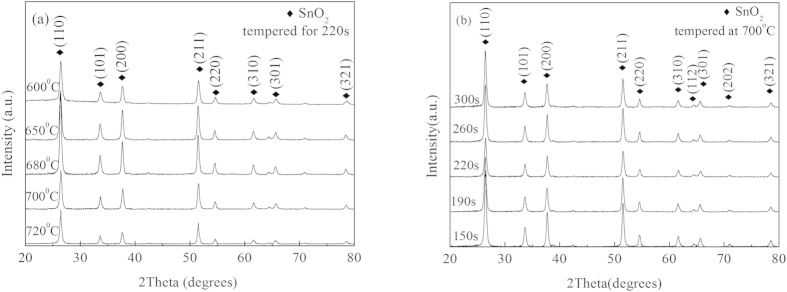
XRD patterns of FTO films tempered at different conditions (a) tempered for 220 s at different temperatures (b) tempered at 700 °C for different time.

XRD patterns (Fig. 1) show that all films tempered at different conditions consist of polycrystalline SnO_2_ with tetragonal structure (P42/mnm (136)) and a high degree of crystallinity. No other phases corresponding to fluoride are detected. The crystal sizes are calculated according to the Debye-Scherer equation, and listed in Table 1. The crystal sizes are in the region of 20.20 ~ 22.54 nm and no obvious variation has been found. Slight changes in the volume of the unit cell of SnO_2_ crystals are observed in the deposited and tempered FTO films (Table 1). The volume is 72.51 ~ 73.00 Å^3^, slightly larger than those of pure SnO_2_with tetragonal structure according to the JCPDS card (71.552 Å^3^). This is probably due to the incorporation of F ions into the O ion sites which are slightly larger than O ions (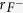 ≈ 1.33 Å, 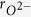 ≈ 1.32 Å).

**Figure 2:**
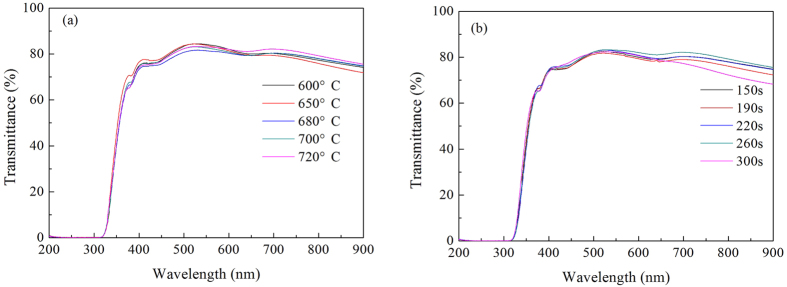
Transmittance spectra of FTO films in the region of 200 ~ 900 nm at different conditions (a) tempered for 220 s at different temperatures (b) tempered at 700 °C for different time.

The optical transmittance spectra of FTO films tempered at different conditions are shown in Fig. 2. The transmittance spectra exhibit a well pronounced fundamental absorption, and there is almost no difference among different conditions. In addition, when tempered at any conditions, the average transmittance values of all the films in the region of 400 ~ 800 nm are about 80%, which reveals that all the tempered FTO films can meet the requirement of high transparency in the visible region for architectural glass and thin-film solar cells.

**Figure 3:**
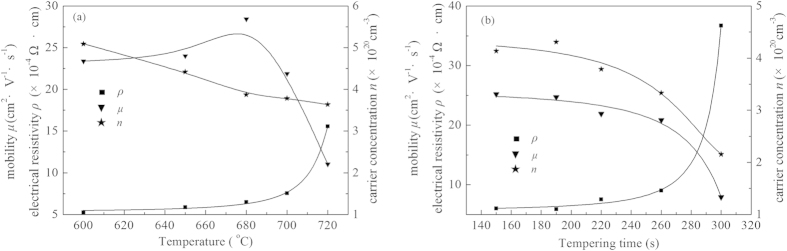
Electrical properties of FTO films tempered at different conditions (a) tempered for 220 s at different temperatures (b) tempered at 700 °C for different time.

The electrical properties (including resistivity, carrier concentration and Hall mobility) of FTO films dependent on different tempering conditions are shown in Fig. 3, and the data are listed in Table 1. It can be seen that all Hall coefficient is negative, which means that all FTO films exhibit n-type conductivity. When the temperature increases from 600 °C to 700 °C, the resistivity increases slowly from 5.25 × 10^−4^ Ω•cm to 7.54 × 10^−4^ Ω•cm. When the temperature reaches 720 °C, the resistivity increases sharply to 15.57 × 10^−4^ Ω•cm. Therefore, the critical tempering temperature of FTO at high temperature is 700 °C. When FTO films tempered at 700 °C for different time, the resistivity increases gradually from 5.99 × 10^−4^ Ω•cm for 150 s to 9.00 × 10^−4^ Ω•cm for 260 s, and then sharply to 36.7 × 10^−4^ Ω•cm for 300 s. Above all, 700 °C and 260 s may be the critical tempering temperature and time, respectively. The reasons for this inference will be discussed below.

The resistivity of FTO films depends on Hall mobility and carrier concentration. It is well known that Hall mobility in doped semiconductors is usually limited by two major scattering mechanisms: grain boundary scattering and ionized impurity scattering^12^. The main scattering mechanism can be deduced from the comparison between the mean free path and the grain size. When the mean free path of free carriers is comparable to the grain size in the films, grain boundary scattering is the dominant mechanism. Based on this, Q. Gao *et al.*^7^ thought that Hall mobility is limited by the grain boundary scattering, because the mean path (13.24 ~ 9.81 nm) in FTO films is comparable to the grain size (about 10 nm). When the mean free path is considerably shorter than the grain size of the films, Hall mobility is limited by the ionized impurity scattering rather than the grain boundary scattering.

The mean free path *l* is calculated according to the following equation^13^:


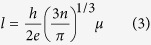


where, *h* is Plank’s constant, *e* is electron charge, *n* is carrier concentration and *μ* is Hall mobility. The mean free path of free carriers in FTO films tempered at different conditions were calculated and are listed in Table 1.

When the temperature is lower than 700 °C for 220 s or the time is less than 260 s at 700 °C, the mean path *l* is in the region of 2.94 ~ 4.04 nm, while the crystal size *D* is in the region of 20.08 ~ 21.60 nm, the ratio of the mean free path and crystal size (*l*/D) is between 13.68% and 18.92%, which reveals that the free path of free carriers is considerably shorter than the grain size of FTO films. Therefore, Hall mobility is mainly limited by ionized impurity scattering, which is in accordance with previously reported results^8^,^14^,^15^. However, when tempered at 720 °C for 220 s or at 700 °C for 300 s, the free path is shortest of 1.60 nm and 0.97 nm, respectively, which is almost three times than the lattice parameters (a = b ≈ 4.76 Å,c ≈ 3.20 Å, in tempered FTO films), indicating that Hall mobility may be limited by lattice vibration, resulting stronger scattering, shorter relaxation time, and lower Hall mobility. Therefore, FTO films tempered at 720 °C for 220 s or at 700 °C for 300 s possesses the lowest mobility.

As seen in Fig. 3, the carrier concentration *n* of FTO films decreases gradually with the increasing temperature at a constant tempering time of 220 s or the increasing time at a constant tempering temperature of 700 °C. It has been proposed theoretically that this carrier concentration depends on oxygen ion vacancies or excess metal ions in FTO films^16^. To investigate this further from the viewpoint of experimental data, XPS was carried out to investigate the stoichiometry of SnO_2_ crystal in FTO films.

**Figure 4:**
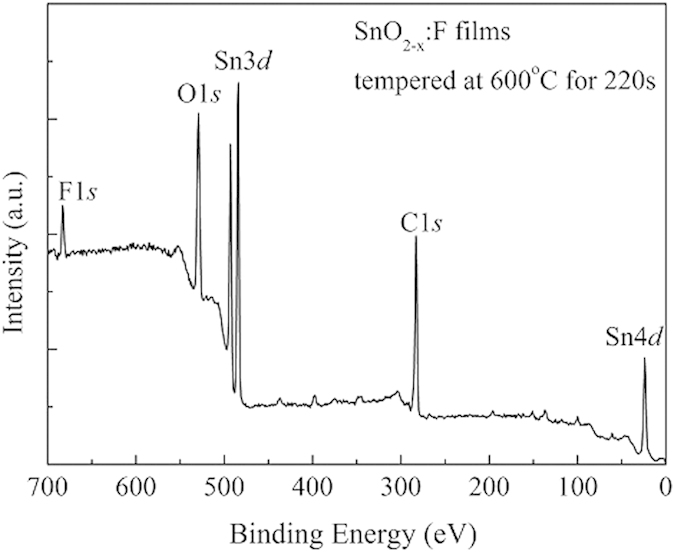
XPS survey spectrum of the surface layer of FTO films tempered at 600 °C for 220 s.

The XPS survey spectrum of film surface tempered at 600 °C for 220 s shows a weak C1*s* peak at about 285.0 eV at the limit of detection (Fig. 4). The main peaks of F1*s*, O1*s*, Sn3*d* and Sn4*d* core levels are well pronounced indicating a high purity of FTO films. Although there is no fluorine phase seen in the XRD patterns of FTO thin films, the element fluorine could be detected using XPS. The molar concentration of fluorine doping in FTO films tempered at different conditions is 4.4 ~ 4.8% calculated according to equation (2) which is in the range that we expected.

**Figure 5:**
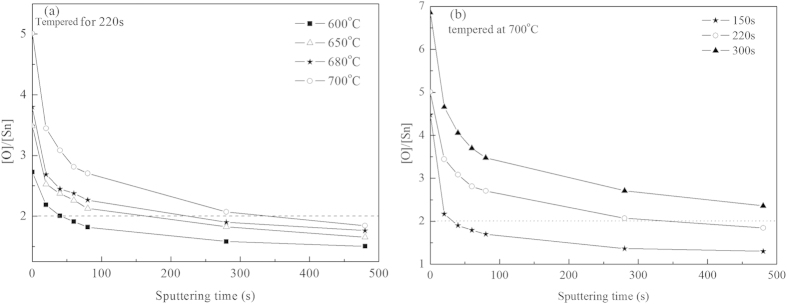
Variation of [O]/[Sn] ratio along the depth of FTO films tempered at different conditions (a) tempered for 220 s at different temperatures (b) tempered at 700 °C for different time.

The relative [O]/[Sn] concentration of FTO films tempered at different conditions during subsequent sputtering are calculated according to equation (2) and shown in Fig. 5. It can be obviously noted that [O]/[Sn] decreases with the sputtering time, independently of tempering conditions of FTO films. The value of [O]/[Sn] at film surface is much higher than the stoichiometric value 2.0 of pure crystalline SnO_2_. Since the microstructure of FTO films prepared by CVD is formed quickly, many defects or dangling bonds exist in the films. According to the previous work^17^,^18^, oxygen exists in three chemical states on the film surface: absorbed oxygen O_abs_ and lattice oxygen of two different types O_I_ and O_II_. Accordingly, if a large amount of oxygen is absorbed on the film surface, it would increase the [O]/[Sn] ratio at the surface layer, as reported previously^17^,^19^. On the other hand, absorbed oxygen could diffuse into deeper layers of the of FTO films resulting in an oxygen gradient from the surface to the center of FTO films. At a certain depth the [O]/[Sn] ratio is lower than 2.0, indicating the presence of lattice oxygen O_I_ in the oxygen-deficient regions, i.e. oxygen vacancies.

In addition, at the same sputtering time, i.e. at the same depth of FTO films, the value of [O]/[Sn] is positively correlated with tempering temperature at a constant tempering time and with tempering time at a constant temperature. D.V. Morgan and co-workers^20^ reported that grain boundary regions in Sn: In_2_O_3_ films acted as pathways for rapid oxygen diffusion into and out of the grains. It can be concluded that O and Sn atoms are redistributed at the diving force of the energy at higher temperature and longer time, more oxygen is allowed to enter the SnO_2_ lattice along grain boundary to compensate for oxygen vacancies created by fluorine doping, then the stoichiometry of SnO_2_ crystal in FTO films is improved, the number of free carriers reduces. Both the lowest mobility and the smallest number of carriers of FTO films when tempered at 720 °C for 220 s or at 700 °C for 300 s leads to the largest resistivity of FTO films.

The calculated emissivity *ε* of the coated glass can be calculated using the sheet resistance *R*_s_ according to the following equations^21^:


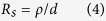



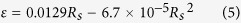


where, *ρ* is the film resistivity and *d* is the film thickness (*d* = 250 nm for FTO films). For the data discussed here, the error of *ε* calculated from equation (5) was below 2%^22^. The values of *ε* are listed in Table 1. FTO films tempered at 700 °C for 260 s possess a relatively proper and low emissivity of 0.38.

## Conclusions

Non-stoichiometric FTO films were deposited by APCVD on a glass substrate coated with a diffusion layer of SiO_x_C_y_. The as-deposited FTO films were tempered at 700 °C for different time and for 220 s at different temperatures, respectively. All the films consist of non-stoichiometric, polycrystalline SnO_2_ with tetragonal structure and a high degree of crystallinity. 700 °C and 260 s may be the critical tempering temperature and time, respectively. When the tempering temperature is higher than 700 °C or the tempering time is longer than 260 s, Hall mobility may be limited by lattice scattering and the number of oxygen vacancies decreases, both the lowest mobility and the smallest number of carriers of FTO films leads to the largest resistivity of FTO films. The molar concentration of fluorine doping in FTO films treated at different conditions was 4.4 ~ 4.8% and confirmed the expectations for this experiment.

## Additional Information

**How to cite this article**: Yang, J. K. *et al.* Reference of Temperature and Time during tempering process for non-stoichiometric FTO films. *Sci. Rep.* **5**, 15001; doi: 10.1038/srep15001 (2015).

## Figures and Tables

**Table 1:** Tempering conditions and the lattice parameters, crystalline size, electrical properties of FTO films.

Tempered conditions		volume of unit cell V (Å^3^)	Crystalline size *D* (nm)	Hall coefficient R_h_(×10^−2^ cm^3^·C^−1^)	mean free path *l*(nm)	*l*/*D*	calculated emissivity *ε*
Temperature	Time						
600 °C		72.596	20.08	−1.23	3.80	18.92%	0.24
650 °C		72.918	20.26	−1.41	3.73	18.41%	0.27
680 °C	220 s	73.002	21.60	−1.85	4.04	18.70%	0.34
700 °C		72.625	21.10	−1.65	3.22	15.26%	0.36
720 °C		72.890	19.65	−1.72	1.60	8.14%	0.54
	150 s	72.509	20.99	−1.51	3.22	15.34%	0.27
	190 s	72.652	21.30	−1.45	3.80	17.84%	0.32
700 °C	220 s	72.625	21.10	−1.65	3.22	15.26%	0.36
	260 s	72.688	21.49	−1.87	2.94	13.68%	0.38
	300 s	72.771	21.11	−2.90	0.97	4.59%	0.45

## References

[b1] Consonni V. *et al.* Preferential orientation of fluorine-doped SnO_2_ thin films: The effects of growth temperature. Acta Mater. 61, 22–31 (2013).

[b2] Yang J. K. *et al.* Structural and optical properties and photoluminescence mechanism of fluorine-doped SnO_2_ films during the annealing process. Acta Mater. 62, 156–161 (2014).

[b3] Hudaya C., Jeon B. J. & Lee J. K. High Thermal Performance of SnO_2_:F Thin Transparent Heaters with Scattered Metal Nanodots. ACS Appl. Mater. Inter. 7, 57–61 (2015). 10.1021/am507497u 25557013

[b4] Renewable Energy World. Review Issue 2004–2005, **7**, 86 (2004).

[b5] Finley J. J. Heat treatment and bending of low-E glass. Thin Solid Films 351, 264–273 (1999).

[b6] Khan A. F., Mehmood M., Aslam M. & Ashraf M. Characteristics of electron beam evaporated nanocrystalline SnO_2_ thin films annealed in air. Appl. Surf. Sci. 256, 2252–2258 (2010).

[b7] Gao Q. *et al.* Effect of glass tempering on microstructure and functional properties of SnO_2_:F thin film prepared by atmosphere pressure chemical vapor deposition. Thin Solid Films 544, 357–361 (2013).

[b8] Yang J. K. *et al.* Studies on the structural and electrical properties of F-doped SnO_2_ film prepared by APCVD. Appl. Surf. Sci. 257, 10499–10502 (2011).

[b9] Zhao H. L., Liu Q. Y., Cai Y. X. & Zhang F. C. Effects of water on the structure and properties of F-doped SnO_2_ films. Mater. Lett. 62, 1294–1296 (2008).

[b10] Kwok R. W. M. XPS Peak Fitting Program for WIN95/98 XPSPEAK Version 4.1, Department of Chemistry, The Chinese University of Hong Kong.

[b11] Wagner C. D., Riggs W. M., Davis L. E., Moulder J. F. & Mnilenberger G. E. Handbook of X-Ray Photoelectron Spectros-copy, Perkin-Elmer, Eden Prairie, MN, 1979.

[b12] Thangaraju B. Structural and electrical studies on highly conducting spray deposited fluorine and antimony doped SnO_2_ thin films from SnCl_2_ precursor. Thin Solid Films 402, 71–78 (2002).

[b13] Noguchi S. & Sakata H. Electrical properties of undoped In_2_O_3_ films prepared by reactive evaporation. J. Phys. D: Appl. Phys. 13, 1129–1133 (1980).

[b14] Lee S. Y., & Park B. O. Structural, electrical and optical characteristics of SnO_2_:Sb thin films by ultrasonic spray pyrolysis. Thin Solid Films 510, 154–158 (2006).

[b15] Agashe C. & Major S. S. Effect of heavy doping in SnO_2_:F films. J. Mater. Sci. 31, 2965–2969 (1996).

[b16] Remes Z., Vanecek M., Yates H. M., Evans P. & Sheel D. W. Optical properties of SnO_2_:F films deposited by atmospheric pressure CVD. Thin Solid Films 517, 6287–6289 (2009).

[b17] Yang J. K., Zhao H. L. & Zhang F. C. Effects of heat treatment on the chemical states of O1s and Sn3d at the surface of SnO_x_:F films by APCVD. Mater. Lett. 90, 37–40 (2013).

[b18] Yang J. K. *et al.* Evolution of element distribution at the interface of FTO/SiO_x_C_y_ films with X-ray photoelectron spectroscopy. Mater. Lett. 133, 247–250 (2014).

[b19] Szuber J., Czempik G., Larciprete R., Koziej D. & Adamowicz B. XPS study of the L-CVD deposited SnO_2_ thin films exposed to oxygen and hydrogen. Thin Solid Films 391, 198–203 (2001).

[b20] Morgan D. V., Aliyu Y. H., Bunce R. W. & Salehi A. Annealing effects on opto-electronic properties of sputtered and thermally evaporated indium-tin-oxide films. Thin Solid Films 312, 268–272 (1998).

[b21] Szczyrbowski J., Dietrich A. & Hartig K. Evaluation and control of the properties of thin sputtered silver films for spectrally selective coatings. Sol. Energy Mater. 16, 103–111 (1987).

[b22] Szczyrbowski J., Dietrich A. & Hartig K. Bendable silver-based low emissivity coating on glass. Sol. Energy Mater. 19, 43–53 (1989).

